# The Effect of *Helicobacter pylori* Infection, Aging, and Consumption of Proton Pump Inhibitor on Fungal Colonization in the Stomach of Dyspeptic Patients

**DOI:** 10.3389/fmicb.2016.00801

**Published:** 2016-05-25

**Authors:** Sadegh Massarrat, Parastoo Saniee, Farideh Siavoshi, Reyhane Mokhtari, Fariborz Mansour-Ghanaei, Saman Khalili-Samani

**Affiliations:** ^1^Endoscopy Department, Digestive Disease Research Institute, Tehran University of Medical SciencesTehran, Iran; ^2^Department of Microbiology, School of Biology, University College of Sciences, University of TehranTehran, Iran; ^3^Gastrointestinal and Liver Diseases Research Center, Guilan University of Medical SciencesRasht, Iran

**Keywords:** *H. pylori*, *C. albicans*, coinfection, gastric diseases

## Abstract

**Background:** The importance of coinfection of *Helicobacter pylori* (*H*.pylori) and *Candida albicans* (*C. albicans*) in the development of gastric diseases is not known. In this study, the frequency of concurrent infection of *H. pylori* and *C. albicans* in dyspeptic patients was assessed while considering age, gender, and PPI consumption of patients.

**Methods:** Gastric biopsies were taken from 74 yeast-positive dyspeptic patients and gastric disease, age, gender, and proton pump inhibitor (PPI) consumption of subjects were recorded. One antral biopsy was used for rapid urease test (RUT) and one for *H. pylori* and yeast cultivation and smear preparation. Bacterial isolates were identified according to spiral morphology and the biochemical characteristics. Yeast isolates were identified on Chromagar and by the Nested-PCR amplification of *C. albicans*-specific topoisomerase II gene. Twenty-seven biopsy smears were Gram-stained and examined by the light microscope for observing *H. pylori* and yeast cells.

**Results:** Fifty-four (73%) of patients were >40 year. Of 68 patients with PPI consumption record, 46 (67.6%) consumed PPI (*p* = 0). Comparison of patients in peptic ulcer group (12, 16.2%) with (6, 8.1%) or without (6, 8.1%) *H. pylori* or in gastritis group (62, 83.8%) with (25, 33.8%) or without (37, 50%) *H. pylori* showed no significant difference (*p* > 0.05). Of the 46 patients who consumed PPI, 13 (17.5%) were *H. pylori*-positive and 33 (44.6%) *H. pylori*-negative (*p* = 0). Ten out of twenty-seven smears showed the occurrence of *H. pylori* cells, including three with yeast cells. Of the 17 *H. pylori*-negative smears, three showed the occurrence of yeast cells only. Yeasts stained Gram-positive or Gram-negative and appeared as single or budding cells.

**Conclusion:** The older age and PPI consumption could favor fungal colonization in the human stomach. The occurrence of a considerable number of *H. pylori*-positive or *H. pylori*-negative patients with gastritis or peptic ulcer shows that co-infection of Candida and *H. pylori* or infection of yeast alone could be associated with dyspeptic diseases. The occurrence of yeast cells in gastric biopsies with different Gram's reactions indicates that fungi might change their cell wall components for establishing a persistent colonization in the stomach.

## Introduction

Yeast colonies were first observed in our lab when gastric biopsies were cultured on selective Brucella blood agar containing antibacterials, for the isolation of *Helicobacter pylori* (*H. pylori*). However, the importance of yeasts, identified as *Candida* species, in the development of gastric diseases was not clear and indeed the isolated yeasts were regarded as the transient colonizers of the human stomach or secondary contaminants (Siavoshi, 1998). This conclusion was drawn from the known fact that an acidic environment of normal human stomach could kill the microbes which enter through oral cavity (Howden and Hunt, 1987; Martinsen et al., 2005; Delgado et al., 2013; Yang et al., 2013; Wang et al., 2014). Accordingly, some investigators described the fungal infection of the stomach as a rare phenomenon with no correlation with gastric diseases (Bearse and Pollock, 1936; Gorbach et al., 1967; Minoli et al., 1984; von Rosenvinge et al., 2013), although others presented the consensus of opinion that fungal infection could lead to gastritis and gastric ulcer and thus should be taken seriously and cured. Consistent with the latter, many investigators reported the isolation of *Candida* yeast from gastric ulcers (Bearse and Pollock, 1936; Kalogeropoulos and Whitehead, 1988; Zwolinska-Wcislo et al., 2001a; Sasaki, 2013) and gastritis cases (Zwolinska-Wcislo et al., 2001b; Kumamoto, 2011). Furthermore, the discovery of *H. pylori* (Marshall and Warren, 1984) and also the recognition of the bacterial genera such as *Streptococcus, Lactobacillus, Propionibacterium*, and *staphylococcus* in the stomach of healthy individuals (Delgado et al., 2013) revealed that the human stomach could serve as a specialized niche for certain microorganisms despite the occurrence of acidic pH and digestive enzymes (Bik et al., 2006; Li et al., 2009; Delgado et al., 2013).

Among published reports, some describe *Candida* yeast as a true microbiota of gastric environment (von Rosenvinge et al., 2013), others as a transient colonizer of gastric epithelium (Karczewska et al., 2009) and in both cases with the potential to become an opportunistic invader that could establish fungemia with severe clinical consequences (Miranda et al., 2009). Indeed, damaged gastric epithelium has been regarded as an important site for fungal spread to vital sites of the human body such as pleural cavity (Ishiguro et al., 2010) or kidney, liver, and spleen (Kennedy and Volz, 1985). Factors favoring fungal colonization in the stomach have been described as old age, malnutrition, intravascular, or bladder catheterization, H2-blocker therapy and the use of wide-spectrum antibiotics (Scott and Jenkins, 1982; Savino et al., 1994). Hypochlorhydria due to atrophic gastritis, gastric surgery or use of proton pump inhibitors (PPIs) could also increase the risk of fungal infection (Goenka et al., 1996; Martinsen et al., 2005). While the clinical significance of fungi in gastric diseases is controversial (Eras et al., 1972) and the need for antifungal therapy has not been reached a consensus (Sasaki, 2013; von Rosenvinge et al., 2013), the etiologic role of *H. pylori* infection in the development of gastritis, peptic ulcers, gastric adenocarcinoma and mucosa-associated lymphoid tissue (MALT) lymphoma has been widely accepted (Suerbaum and Michetti, 2002) and antimicrobial therapy measurements have been designed (Lam and Talley, 1998; Malfertheiner et al., 2012).

Several studies have reported *Candida* yeast and *H. pylori* as the inhabitants of the human stomach (Kalogeropoulos and Whitehead, 1988; Zwolinska-Wcislo et al., 2001a; Karczewska et al., 2009); however, the importance of their mutual interaction in the development of gastric diseases such as gastritis and peptic ulcers is not clearly understood. It is not known whether the concomitant infection of *Candida albicans* (*C. albicans*) and *H. pylori* in the stomach could exacerbate the clinical outcome compared with the infection of each microorganism alone. Indeed, a considerable number of studies have described the implication of yeast in gastric diseases through microscopic observations of the yeast cells in the stained histopathology slides of gastric biopsies, especially in *H. pylori*-negative patients (Sari et al., 2003; Jung et al., 2009; Sasaki, 2013). In this study, 74 dyspeptic patients with gastric biopsies positive for the growth of *Candida* yeast were recruited. Patients were classified into gastritis and peptic ulcer groups, with or without *H. pylori* infection. The frequency of the concurrence of yeast and *H. pylori* infection was assessed while considering PPI consumption and age and gender of patients.

## Methods

### Patients

From 530 dyspeptic referrals to the endoscopy unit of Shariati hospital, Tehran-Iran, within 2 years (2012–2014), 74 (14%) which were yeast-positive were recruited in this study. Patients included 36 (48.6%) males and 38 (51.4%) females with the mean age of 50 ± 15, 54 (73%) >40 year and 20 < 40 year. Patients were classified into gastritis and peptic ulcer groups according to endoscopic findings. The status of *H. pylori* infection and PPI consumption were recorded for each patient. All patients signed an informed consent, and the study was approved by the research ethics committee of Tehran University of Medical Sciences.

### Rapid urease test (RUT), isolation of *H. pylori* and yeast and smear examination

Two antral biopsies were taken from each dyspeptic patient. One biopsy was used for RUT and the other for cultivation of *H. pylori* and yeast. Preparation of RUT solution and performance of the test was according to manufacturer's instruction (Bahar afshan Co., Iran). Briefly, gastric biopsies were put into urea solution (pH 6.8) and the color change from yellow to pink, within 24 h, was recorded as positive. For *H. pylori* and yeast cultivation, Brucella blood agar containing 7–10% defibrinated sheep blood and antibacterials; vancomycin (10 mg/L), trimethoprim (5 mg/L), and polymixin B (50 μg/L) was used for surface-inoculation of gastric biopsies. After culturing, the remaining of each biopsy was smeared on the glass slide, Gram-stained and examined by the light microscopy for observing *H. pylori* and yeast cells. Cultured plates were incubated in the CO_2_ incubator at 37°C and examined after 2–5 days for observing bacterial and yeast colonies. The identity of bacterial isolates as *H. pylori* was confirmed by spiral morphology and positive results of catalase, oxidase, and urease tests. Biopsies were determined as *H. pylori*-positive if the result of one test; RUT or culture showed *H. pylori* occurrence.

### Identification of yeast isolates

All the 74 yeast isolates were identified as *C. albicans* according to their interaction on Chromagar (Chromagar, France) and production of green colonies. The identity of yeasts colonies was confirmed by the Nested-PCR amplification of *C. albicans*—specific topoisomerase II gene according to Kanbe et al. study (Kanbe et al., 2002). The first-step PCR was performed using a set of degenerate primers (F: 5′-GGTGGWMGDAAYGGDTWYGGYGC-3′ and R: 5′-CRTCNTGATCYTGATCBGYCAT-3′). The amplified product was then used for the second PCR amplification using the primers F: 5′-TTGAACATCTCCAGTTTCAAAGGT-3′ and R: 5′-AGCTAAATTCATAGCAGAAAGC-3′. Electrophoresis was performed for observing the amplicons with the size of 665 bp.

### Statistical analysis

Statistical analysis was performed to find the correlation between age, gender or PPI consumption and *H. pylori* or fungal colonization in the stomach. Further analysis was performed to determine the probability of the consistency of positive or negative results of culture and RUT. Non-parametric methods used for data analysis were Chi-square and Fisher's Exact tests with the level of significance *p* < 0.05. Data were collected and analyzed using SPSS version 19.

## Results

Among the 74 yeast-positive patients, 36 (48.6%) were males and 38 (51.4%) females with no significant difference (*p* > 0.05). Fifty-four (73%) were >40 year of age and 20 < 40 year of age, with a significant difference (*p* = 0). Among 36 males, the number of patients with age >40 year (30, 83.3%) was higher than those with age < 40 year (6, 16.7%), showing a significant difference (*p* = 0). However, among 38 females, 24 (63.2%) were >40 year of age and 14 (36.8%) < 40 year of age, with a considerable difference but not significant (*p* > 0.05). Out of 68 patients whose PPI consumption was recorded, 46 (62.1%) consumed PPI and the remaining 22 (29.8%) did not, showing a significant difference (*P* = 0). Thirty-one out of the 74 patients (41.9%) were *H. pylori*-positive (15 males and 16 females). Twenty (27%) were >40 year of age and 11 (14.9%) < 40 year of age (*p* > 0.05). The remaining 43 (58.1%) were *H. pylori*-negative (21 males and 22 females); 34 (45.9%) >40 year of age and 9 (12.2%) < 40 year of age (*p* = 0). Patients were classified into two groups; 12 (16.2%) with ulcer and 62 (83.8%) with gastritis. Out of the 12 ulcer patients; 6 (8.1%) were *H. pylori*-positive and 6 (8.1%) *H. pylori*-negative (*p* > 0.05). Among the 62 gastritis patients, 25 (33.8%) were *H. pylori*-positive and 37(50%) *H. pylori*-negative (*p* > 0.05). Out of the 46 patients who consumed PPI, 13 (17.5%) were *H. pylori*-positive and 33 (44.6%) *H. pylori*-negative (*p* = 0). The remaining 22 (29.8%) patients without PPI consumption included 15 (20.3%) *H. pylori*-positive and 7 (9.5%) *H. pylori*-negative (*p* > 0.05; Table 1).

**Table 1:** **Classification of 74 yeast-positive patients according to gastric disease, the occurrence of ***H. pylori*** infection and PPI consumption**.

	**Gastric disease**		**PPI consumption**		
	**Gastritis (%)**	**Ulcer (%)**	**Yes (%)**	**No (%)**	**No data available (%)**
***H. pylori***					
Positive	25 (33.8)	6 (8.1)	13 (17.5)	15 (20.3)	3 (4.05)
Negative	37 (50.0)	6 (8.1)	33 (44.6)	7 (9.5)	3 (4.05)
Total 74 (100%)	62 (83.8)	12 (16.2)	46 (62.1)	22 (29.8)	6 (8.1)

**Figure 1:**
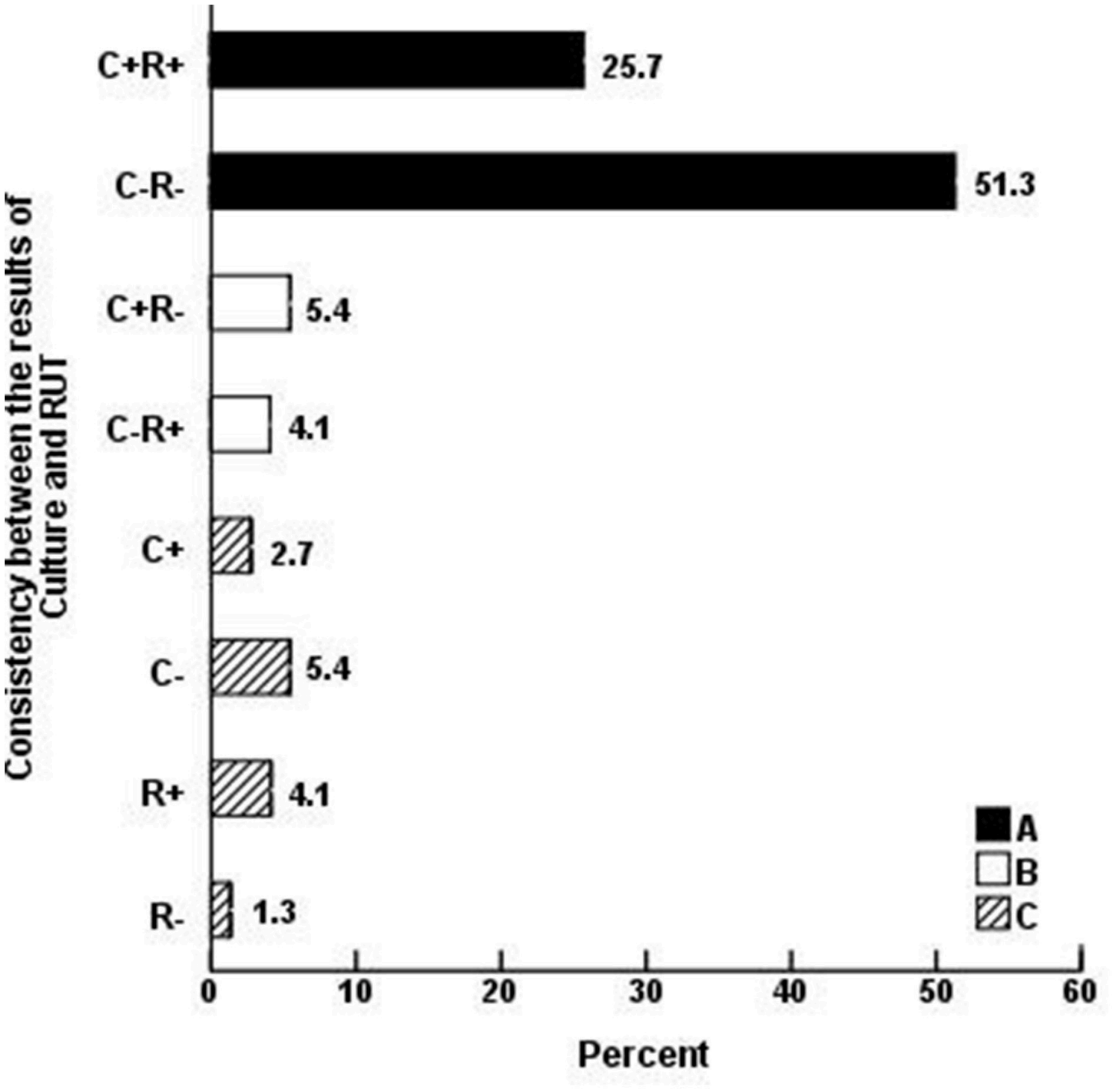
**Consistency between the results of ***H. pylori*** culture (C) and RUT (R) in 74 gastric biopsies**. The frequency of biopsies with consistency in the results of RUT and culture (**A:** 57, 77%), including both positive (19, 25.7%) and both negative (38, 51.3%) was higher than those without consistency in the results of two tests (**B:** 7, 9.5%), including 4 (5.4%) culture-positive and 3 (4.1%) RUT-positive (*p* = 0). The remaining (**C:** 10, 13.5%) biopsies included 2 (2.7%) culture-positive, 4 (5.4%) culture-negative, 3 (4.1%) RUT-positive, and 1(1.3%) RUT-negative.

Seventy-four biopsies were classified according to the results of culture and RUT and 27 according to the results of the microscopic examination of gastric biopsies smears (Table 2). Statistical analyses showed that the frequency of biopsies with consistency in the results of RUT and culture (A: 57, 77%), including both positive (19, 25.7%) and both negative (38, 51.3%) was higher than those without consistency in the results of two tests (B: 7, 9.5%), including 3 (4.1%) RUT-positive and 4 (5.4%) culture -positive (*p* = 0). The remaining biopsies (C:10, 13.5%) included 2 (2.7%) culture- positive, 4 (5.4%) culture- negative, 3(4.1%) RUT- positive and 1(1.3%) RUT-negative (Table 2, Figure 1).

**Table 2:** **The frequency of positive or negative results of culture and RUT of 74 gastric biopsies and microscopic observation of 27 gastric biopsies**.

**Diagnostic tests**	**Results**														
Culture	+	+	+	−	−	−	+	+	−	−	+	−	N	N	N
RUT	+	+	+	−	−	−	−	−	+	+	N	N	+	+	−
Smear	+	−	N	−	+	N	−	N	+	N	N	N	+	−	N
Frequency	6	9	4	4	1	33	3	1	1	2	2	4	2	1	1
Total 74 (100%)	57 (77.0%)						7 (9.5%)				10 (13.5%)				

*N: no data available*.

**Figure 2:**
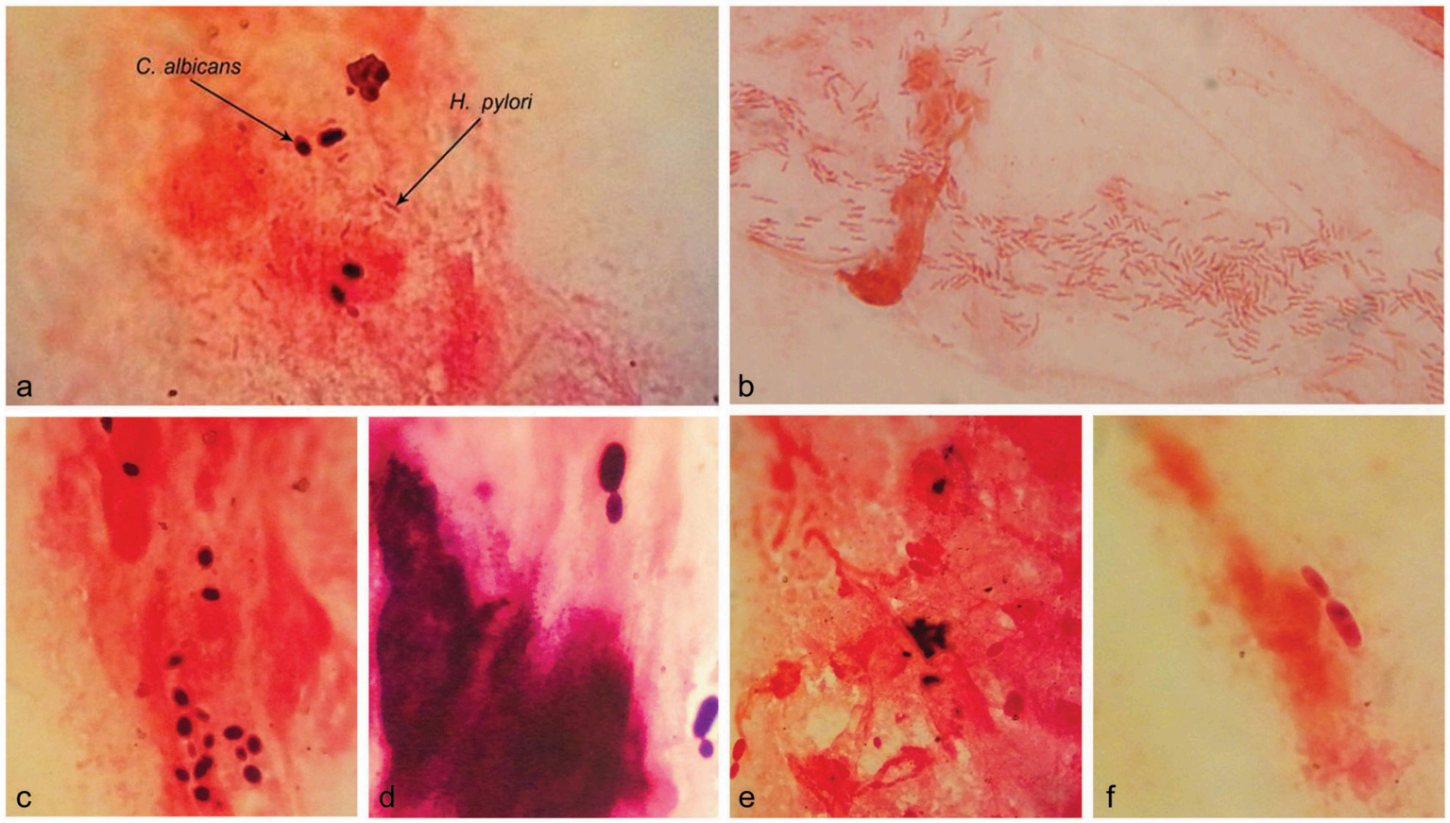
**Microscopic observation of ***H. pylori*** and ***Candida*** yeast in the Gram-stained smears of gastric biopsies from patients with gastritis**. The Concurrence of *H. pylori* and *Candida* yeast cells **(A)**. The spiral cells of *H. pylori* **(B)**. Yeast cells; Gram positive **(C)** or Gram-negative **(E)** and some in the stage of budding **(D,F)**. Original magnification: X 1250.

Examination of 27 Gram-stained biopsy smears by light microscopy revealed the presence of *H. pylori* cells in 10, including three with the concomitant occurrence of *H. pylori* and yeast cells. The three biopsies were culture- and RUT-positive and belonged to two patients with gastritis and one with peptic ulcer. The remaining 17 biopsy smears were negative for the presence of *H. pylori*, but three showed the presence of yeast cells. The three biopsies were culture- and RUT-negative and belonged to the patients with gastritis. The six yeast-positive biopsies were taken from patients with no record of PPI consumption (Tables 1, 2). In microscopic observations, yeast cells appeared as single cells, some in the stage of budding. Moreover, some yeast cells appeared Gram-positive and some Gram-negative (Figure 2).

## Discussion

*Candida* yeast (Whyte et al., 1982; Sharp et al., 1992) and *H. pylori* (Webb et al., 1994; Malaty et al., 2002) colonize the human gastrointestinal tract early in life. It appears that both microorganisms are well-adapted to establish in the specialized environment of the human stomach (Kalogeropoulos and Whitehead, 1988; Zwolinska-Wcislo et al., 2001a; Loster et al., 2006; Karczewska et al., 2009). Detection of either *H. pylori* or *Candida* yeast in gastric biopsies of dyspeptic patients indicates the implication of these two microorganisms in gastric diseases. However, the relationship between their concomitant occurrence and gastric diseases has not been thoroughly investigated.

Out of the 74 yeast-positive patients, 36 (48.6%) were males and 38 (51.4%) females with the mean age of 50 ± 15, 54 (73%) were >40 year of age and 20 < 40 year of age, with a significant difference (*p* = 0). Forty six out of 68 patients (67.6%) consumed PPI and 22 (32.4%) did not, showing a significant difference (*p* = 0). These results indicate that both the older age and PPI consumption could be important risk factors in favor of fungal colonization. *Candida* spp. have been isolated from gastric biopsies, the brush samples of gastric mucosa and peritoneal fluid and the frequency of fungal isolation was higher in older patients (Morishita et al., 1993; Goenka et al., 1996) or in those with hypochlorhydria (Goenka et al., 1996). Furthermore, among the 178 patients with benign gastric ulcer, there was a significant difference between the number of older patients at a mean age of 64.2 year with yeast in their histopathology preparation (36, 20.2%) and those with the mean age of 56.2 year without yeast (142, 79.8%; *p* < 0.01). It was concluded that the older age was the only risk factor for fungal infection, and gender, cigarette smoking and tea or coffee consumption were not implicated in the development of gastric ulcer (Wu et al., 1995). It appears that immunosuppression due to aging could facilitate fungal invasion of the gastric ulcer (Neeman et al., 1981; Loffeld et al., 1988). Reports also indicate that hypochlorhydria provides favorable conditions for the growth of fungi (Goenka et al., 1996; Martinsen et al., 2005) and bacteria (Thorens et al., 1996) in the stomach. Statistical analysis showed a high consistency between the positive or negative results of *H. pylori* culture and RUT. When comparing the *H. pylori*-positive (31, 41.9%) with *H. pylori*-negative (43, 58.1%) patients, it was found that age, gender, and PPI consumption are not the important risk factors for *H. pylori* infection.

Among the 74 yeast-positive patients, 12 (16.2%) had peptic ulcer and 62 (83.8%) chronic gastritis. While reports indicate the fungal colonization in the gastric mucosa of 7–33% asymptomatic patients (Minoli et al., 1982; Loffeld et al., 1988), the fungal infection in the gastric ulcers has been estimated as 6.9% (Di Febo et al., 1985) to 33.3% (Katzenstein and Maksem, 1979) and 36% (Gotlieb-Jensen and Andersen, 1983). In ulcer group, the number of *H. pylori*-positive patients (6, 8.1%) and *H. pylori*-negative patients (6, 8.1%) was similar (*p* > 0.05). The same results were observed in gastritis group; 26 (35.1%) *H. pylori*-positive and 36 (48.7%) *H. pylori*-negative, with no significant difference (*p* > 0.05). These results demonstrate that a considerable number of *H. pylori*-negative patients also suffered from gastric ulcer or gastritis. Accordingly, the concurrence of *Candida* yeast and *H. pylori* or yeast infection alone could increase the risk of development of chronic gastritis or peptic ulcer. In a study on 293 biopsies of dyspeptic patients RUT, culture and microscopy were used to demonstrate the concurrence of fungal cells with *H. pylori* in 14% of gastric ulcer patients, 4% of those with chronic gastritis and 2% of control subjects (Zwolinska-Wcislo et al., 2001a). In another study on 158 patients, the co-existence of *H. pylori* and *Candida* yeast was found in 18 (11%) and was more frequent in patients with gastric ulcer (Karczewska et al., 2009). It has been proposed that the establishment of *H. pylori* in gastric epithelium might play an important role in the fungal colonization (Zwolińska-Wcisło et al., 2003). Furthermore, the coexistence of yeast and *H. pylori* in the stomach might exacerbate the inflammatory response and tissue damage (Diebel et al., 1999; Zwolinska-Wcislo et al., 2001a; Karczewska et al., 2009).

Microscopic examination of the 27 Gram-stained smears of gastric biopsies showed the presence of yeast cells in six (8.1%) specimens, three concomitant with *H. pylori*. Yeasts appeared as single cells which stained Gram-positive or Gram-negative and some in the stage of budding. A report showed microscopic observation of both *Candida* yeast and *Campylobacter*-like organisms in 12% of gastric biopsies from patients with gastric ulcer (Kalogeropoulos and Whitehead, 1988). Previous studies, dating back to 1936 or earlier, demonstrated that microscopic observation of fungi in biopsy specimens from gastric ulcer is an important indication of disease. It was proposed that fungi typical oval morphology might change to hyphae when colonizing the tissues (Bearse and Pollock, 1936). However, more recent reports proposed that most dimorphic fungi that are human pathogens, invade tissues by budding and form filamentous hyphae in the external environment (Gow et al., 2002). Investigators believe that fungi have learned to establish on the mucosal surfaces of the human body by minimizing the induction of host immune response (Hube, 2009). They do this by changing the proteins and carbohydrates of their cell wall which determine the fungal morphology as yeast or hyphae. Accordingly, components of the cell wall and thus morphology play an important role in the fungal establishment in the host (Gow et al., 2002; Gow and Hube, 2012). In the present study, the occurrence of yeast cells in the biopsy smears, with different reactions to Gram's stain, might reflect changes in the cell wall components that help the persistent colonization of yeast cells in the stomach.

Results of this study demonstrate that both the older age and PPI consumption are among the risk factors for fungal colonization in the stomach. Furthermore, the concurrence of *Candida* yeast and *H. pylori* or yeast infection alone could be involved in the development of peptic diseases. Considering the well-known role of *H. pylori* and less-recognized importance of *Candida* yeast in gastric diseases, the possible role of the concurrent infection of these two microorganisms in the development of chronic gastritis and peptic ulcer warrants in depth investigations (Zwolinska-Wcislo et al., 2001a). Furthermore, the widely accepted role of *H. pylori* in gastric cancer (Malfertheiner et al., 2012) and the frequent detection of fungal infection in patients with gastric cancer (Sasaki, 2013) indicate that the mutual interaction of *Candida* yeast with *H. pylori* might go much further, leading to the development of gastric malignancies (Wang et al., 2014). Concomitant colonization of *H. pylori* and *Candida* yeast in the human gastric niche might refer to their evolutionary association which begun long time ago and has led to the endosymbiotic relationship of *H. pylori* with *Candida* yeast (Siavoshi et al., 2005; Siavoshi and Saniee, 2014). Future studies will elucidate the crucial and determinant role of these two microorganisms in human health and disease.

## Author contributions

SM, PS, and FS designed the experiment. FM provided the gastric biopsies. RM did part of the research work. FS and PS did part of the lab work. SK did the graphics and tables. FS and PS wrote the paper.

### Conflict of interest statement

The authors declare that the research was conducted in the absence of any commercial or financial relationships that could be construed as a potential conflict of interest.
